# Make No Bones about It: Long Bones Scale Isometrically

**DOI:** 10.1371/journal.pbio.1002211

**Published:** 2015-08-04

**Authors:** Caitlin Sedwick

**Affiliations:** Freelance Science Writer, San Diego, California, United States of America

## Abstract

Long bones are far from being simple cylinders, so how is the relative positioning of their various features maintained during growth? A new study shows that growth is isometric and that drift from the correct position is minimized. Read the Research Article.

A common developmental problem faced by growing multicellular organisms is one of scaling: as the body grows, organs must retain the proper proportions to function well. Baby-sized lungs could not obtain enough oxygen and a baby-sized heart would pump insufficient blood to supply the needs of an adult, so these organs must scale up in size as the organism grows. Limbs must also scale in both length and diameter to support the growing body mass, although the proportional relationship of limb length to body mass will change throughout growth. However, unlike soft tissue organs, which may expand across their entire volumes, vertebrate long bones lengthen only at specialized sites located at their longitudinal ends, where cartilaginous tissue is gradually ossified. The proteins and pathways that drive growth at these sites are well studied, but how they govern proportional scaling of the bone throughout development is poorly understood. Studying bone morphogenesis may therefore bring useful insights into the timing and operation of the developmental signals that guide bone growth.

If long bones were simple cylinders, then their growth could be easily conceptualized as taking place through gradual lengthening when new material is added at either end. But actually, the bones sport various protuberances and ridges—symmetry-breaking elements—that allow attachments to the musculature and formation of joint structures. The placement of these elements is crucial for locomotion; therefore, it’s necessary to consider the positioning of these elements during bone morphogenesis. In this regard, scientists have suggested that bone morphogenesis could be managed through either isometric or allometric scaling processes. If bones employ isometric scaling, then the relative position of symmetry-breaking elements would be fixed throughout development; a protuberance that first appears at a spot 20% down the length of the embryonic bone would remain at approximately 20% as the bone grows. By contrast, with allometric scaling that element’s relative positioning could change dramatically as the organism grows. Which type of scaling is employed in long bone growth has never been examined in depth, but Tomer Stern, Elazar Zelzer, and colleagues have now made great strides with this problem in their paper published in *PLOS Biology*.

To determine whether scaling during bone growth is isometric or allometric, Stern and colleagues assembled a large database containing 3-D micro-CT images of mouse long bones at different stages of development from embryo to adult. Comparing the position of symmetry-breaking elements for each bone revealed that, with only a few exceptions, the relative positions of the elements on a given bone remain the same throughout growth (Fig 1, bottom panel). Therefore, the authors conclude, long bone growth is mostly isometric.

**Fig 1:**
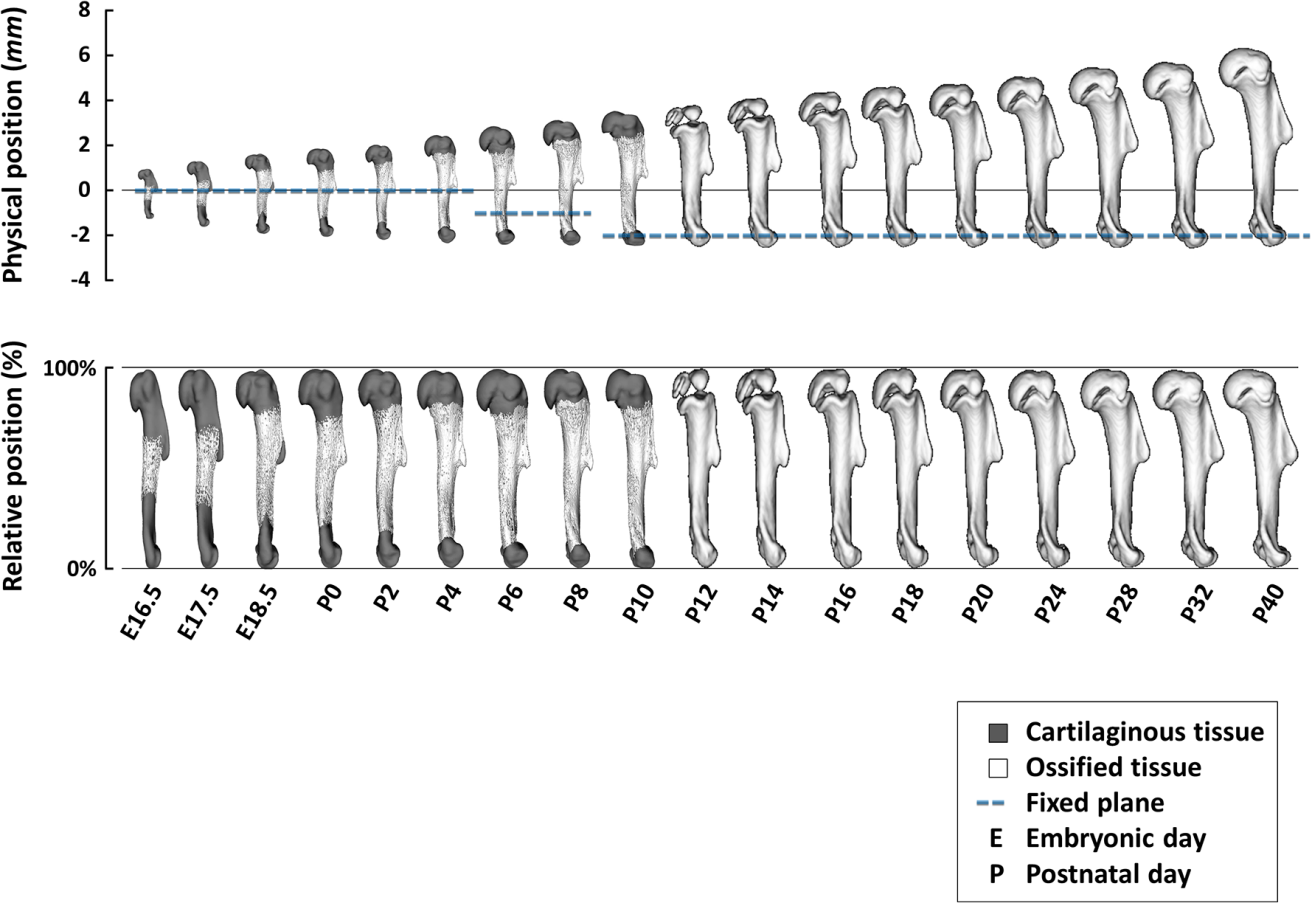
Developing long bones accurately maintain their body proportions during more than a 5-fold increase in length in a cost-optimal manner by regulating the balance between the growth rates at their two ends. *Image credit: Tomer Stern*.

Yet if long bone growth is isometric, how is the relative positioning of symmetry-breaking elements maintained as bones lengthen at their ends? Bones are rigid structures made up of mineralized tissue, thus element positioning cannot be adjusted simply by moving cells about, as could occur in soft tissue. However, bone can be modeled and modeled; existing bone can be dissolved by osteoclasts and new bone added by osteoblasts. It has been proposed that such a mechanism could be used to “drift” elements so as to preserve their relative positions during growth. Therefore, Stern et al. set out to develop an algorithm to test whether bone elements drift during isometric growth.

Unexpectedly, the authors’ analysis showed that, while a few elements do drift, the rest do not. In fact, the researchers found that for each bone, a transverse plane can be drawn at the location where the ratio of the plane’s distance to either end equals the ratio of growth rates at the respective ends (Fig 1, top panel). This “fixed plane” always falls nearby the non-drifting elements, and only the elements that are significantly distant from this plane show evidence of drift. However, the location of the fixed plane, and therefore an element’s relationship to it—which predicts the amount of drift needed to maintain the element’s relative position on the bone—will shift during development if the ratio of growth rates at the ends change.

Stern and colleagues explain that relative positioning of most symmetry-breaking elements is preserved because of their proximity to the fixed plane. The fixed plane shifts infrequently, so these elements rarely need to drift; only elements far from the fixed plane need drift very much. This minimizes the energetic investment needed to resorb and regrow elements, then reposition their associated muscles or tendons during bone growth. The authors speculate that the connective tissue that surrounds the growing bone might regulate relative growth rates at the two bone ends by secreting signaling molecules, either according to a predetermined genetic program, or in response to tension generated by nearby muscle and ligament attachments. It will be interesting to see future work elaborate upon the molecular and cellular details of this mechanism.
